# Diagnostic Performance of Non-invasive Tests for Hepatic Fibrosis Using Liver Biopsy As the Reference Standard: A Systematic Review and Meta-Analysis

**DOI:** 10.7759/cureus.110459

**Published:** 2026-06-08

**Authors:** Sri Lakshmi Kothakapa, Sulekha Ramireddy, Sanjay Reddy Thanugundla, Sainithya Chittireddy, Pruthvi Raj Nenavath, Aarti Prodduku, Charvika Lakavath, Neha Poloju, Naga Teja Thota, Prannav koolla, Harika Koduri, Muralidhar Chinnapaka

**Affiliations:** 1 Internal Medicine, Malla Reddy Institute of Medical Sciences, Hyderabad, IND; 2 Internal Medicine, Prathima Institute of Medical Sciences Karimnagar, Hyderabad, IND; 3 Pharmacology, Government Medical College Maheshwaram, Hyderabad, IND

**Keywords:** apri, diagnostic accuracy, fib-4, liver biopsy, liver fibrosis, magnetic resonance elastography, meta-analysis, transient elastography

## Abstract

Liver biopsy remains the reference standard for staging hepatic fibrosis, but its invasive nature, sampling error, cost, and possible complications have encouraged wider use of non-invasive tests. This systematic review and meta-analysis compared serum-based indices and elastography methods with liver biopsy for detecting fibrosis in chronic liver disease (CLD). A literature search was completed in March 2026 using PubMed/MEDLINE, Embase, Scopus, Web of Science, Cochrane Library, and Google Scholar. Eligible diagnostic accuracy studies assessed serum-based markers, including aspartate aminotransferase to platelet ratio index (APRI), fibrosis-4 index (FIB-4), fibrotest/enhanced liver fibrosis (ELF) panels, and imaging-based modalities, including transient elastography (TE), shear wave elastography (SWE), and magnetic resonance elastography (MRE), using liver biopsy as the reference standard. Pooled sensitivity, specificity, diagnostic odds ratio (DOR), and summary receiver operating characteristic (SROC) area under the curve (AUC) were calculated using a random-effects model in Stata version 18.0 (StataCorp LLC, College Station, TX), and study quality was assessed with Quality Assessment of Diagnostic Accuracy Studies-2 (QUADAS-2).

Forty-two studies, including 12,486 patients, were analyzed. For significant fibrosis, TE showed a pooled sensitivity of 84.6%, a specificity of 79.8%, and an area under the curve (AUC) of 0.88, while FIB-4 and APRI showed moderate accuracy with AUC values of 0.79 and 0.75, respectively. For advanced fibrosis, TE showed a sensitivity of 87.2%, a specificity of 82.6%, a DOR of 31.8, and an AUC of 0.91. Magnetic resonance elastography had the best overall performance, with AUC values of 0.92 for significant fibrosis, 0.94 for advanced fibrosis, and 0.96 for cirrhosis. Accuracy increased with fibrosis severity, although heterogeneity was seen due to differences in disease aetiology, cut-off values, and biopsy quality. Overall, elastography-based tests are reliable for advanced fibrosis and cirrhosis, while APRI and FIB-4 remain useful, low-cost first-line screening tools. A stepwise approach using serum scores followed by elastography may help reduce unnecessary biopsies without compromising diagnostic confidence.

## Introduction and background

Hepatic fibrosis is a progressive wound-healing response to chronic or repeated liver injury. It is characterized by activation of hepatic stellate cells and excess deposition of extracellular matrix, which gradually distorts normal liver architecture and replaces functional hepatic tissue with scar tissue [1-4]. Although early fibrosis may remain clinically silent, progression can result in portal hypertension, hepatic decompensation, hepatocellular carcinoma, liver transplantation, and liver-related mortality [3-5]. Therefore, accurate assessment of the fibrosis stage is central to risk stratification, treatment planning, surveillance, and long-term follow-up in patients with chronic liver disease (CLD).

Chronic liver disease may result from several causes, including chronic hepatitis B and C, alcohol-associated liver disease, metabolic-dysfunction-associated steatotic liver disease (MASLD), autoimmune liver disease, and cholestatic disorders [4, 5]. Irrespective of the underlying etiology, the fibrosis stage remains one of the strongest predictors of clinical outcome. In clinical practice, fibrosis assessment should identify not only the presence of fibrosis but also clinically meaningful thresholds such as significant fibrosis, advanced fibrosis, and cirrhosis. Significant fibrosis generally indicates a stage at which closer monitoring and disease-specific treatment become important; advanced fibrosis identifies patients at higher risk of progression and complications, and cirrhosis marks the stage requiring surveillance for varices, liver failure, and hepatocellular carcinoma [6-8].

Liver biopsy has traditionally been regarded as the reference standard for staging hepatic fibrosis. It allows direct histological assessment of inflammation, steatosis, ballooning, cholestasis, architectural distortion, and fibrosis patterns [6-10]. Histological scoring systems such as meta-analysis of histological data in viral hepatitis (METAVIR), Ishak, Brunt, and the non-alcoholic fatty liver disease (NAFLD) Activity score have helped standardize fibrosis reporting in clinical studies [6-8]. However, a biopsy has several limitations. It is invasive, relatively costly, uncomfortable for patients, and unsuitable for repeated monitoring in many clinical situations. It also carries a small but definite risk of bleeding and other procedure-related complications [9,10]. In addition, sampling error can occur because the biopsy core represents only a small portion of the liver, and fibrosis may be unevenly distributed [11-13]. Specimen length, number of portal tracts, tissue quality, and interobserver variation may further affect staging accuracy [12,13].

These limitations have increased the clinical need for safer, repeatable, and accessible non-invasive methods for fibrosis assessment. Current European Association for the Study of the Liver (EASL) guidance also supports the use of non-invasive tests for assessing liver disease severity and prognosis across different CLD etiologies [14,15]. Non-invasive tests broadly include serum-based indices, combined biomarker panels, and imaging-based stiffness measurements. Simple serum scores such as the aspartate aminotransferase to platelet ratio index (APRI) and fibrosis-4 (FIB-4) index are widely used because they are inexpensive and based on routinely available laboratory parameters [16,17]. The APRI uses aspartate aminotransferase and platelet count, while FIB-4 combines age, aspartate aminotransferase, alanine aminotransferase, and platelet count [16,17]. Other indices, including the NAFLD fibrosis score and Forns index, are also used for risk classification in selected clinical settings [18,19].

Direct and combined serum panels, such as hyaluronic acid, tissue inhibitor of metalloproteinases, procollagen III N-terminal peptide, FibroTest, ELF test, and the hepascore, aim to reflect extracellular matrix turnover more directly [20-23]. These tests may offer better diagnostic performance than simple indirect scores in some settings, but their use may be limited by cost, availability, and the need for standardized laboratory platforms, particularly in resource-limited centers. Imaging-based methods estimate liver stiffness as an indirect marker of fibrosis. Transient elastography (TE) is the most widely studied technique and has been evaluated against liver biopsy in several CLD populations [24-30]. It is rapid, painless, repeatable, and suitable for outpatient use. Other modalities, including acoustic radiation force impulse (ARFI) imaging, point shear wave elastography (SWE), two-dimensional SWE, and magnetic resonance elastography (MRE), have further expanded the role of non-invasive fibrosis assessment [29-31].

Despite their advantages, non-invasive tests have important limitations. Serum scores may be affected by acute hepatitis, hemolysis, non-hepatic thrombocytopenia, alcohol intake, systemic inflammation, renal dysfunction, and age [16-18,31-36]. Elastography may overestimate fibrosis in active inflammation, cholestasis, or hepatic congestion or after food intake and may be technically limited in obesity, ascites, or narrow intercostal spaces [27,28,31]. Most non-invasive tests perform better for excluding advanced fibrosis or identifying cirrhosis than for distinguishing intermediate stages. This creates a diagnostic grey zone where combined testing or liver biopsy may still be required.

A stepwise diagnostic approach has therefore been proposed, using simple serum markers as first-line screening tools followed by elastography in patients with indeterminate or high-risk results [14,15,24,32-34]. Such an approach may reduce unnecessary biopsies while maintaining reasonable diagnostic confidence. However, reported accuracy varies across studies because of differences in disease etiology, fibrosis thresholds, biopsy quality, scoring systems, cut-off values, study population, and statistical methods [24,29,30,33,37-40]. The increasing burden of MASLD has further strengthened the need for reliable, scalable, and clinically practical non-invasive fibrosis assessment [41-46].

Previous meta-analyses and guideline-based reviews have assessed selected non-invasive tests, but direct comparison across multiple serum-based markers, combined biomarker panels, and elastography-based techniques remains clinically useful, particularly when liver biopsy is used as the common reference standard. The present systematic review and meta-analysis were conducted to evaluate the diagnostic accuracy of commonly used non-invasive tests for hepatic fibrosis using liver biopsy as the reference standard. It focuses on clinically relevant fibrosis thresholds, namely significant fibrosis, advanced fibrosis, and cirrhosis, and compares the performance of serum-based indices, combined biomarker panels, and imaging-based techniques. This updated synthesis may help support practical diagnostic pathways that balance accuracy, safety, cost, availability, and feasibility in routine clinical care.

## Review

Methods

Study Design and Reporting Framework

This systematic review and meta-analysis were conducted using a predefined methodological approach, including clearly framed eligibility criteria, systematic literature search, structured study selection, standardized data extraction, QUADAS-2-based quality assessment, and planned statistical synthesis. The review was reported in accordance with the Preferred Reporting Items for Systematic reviews and Meta-Analyses (PRISMA) [44] and the PRISMA extension for diagnostic test accuracy (DTA) [47] recommendations to ensure transparency, reproducibility, and methodological clarity.

The PRISMA flow diagram presents the study selection process from identification to final inclusion. A total of 1,284 records were retrieved from database searches, and 76 additional records were identified through reference lists and manual searching. After merging all sources, 1,360 records were screened for duplicates. Of these, 312 duplicate records were removed, leaving 1,048 records for title and abstract screening.

**Figure 1:**
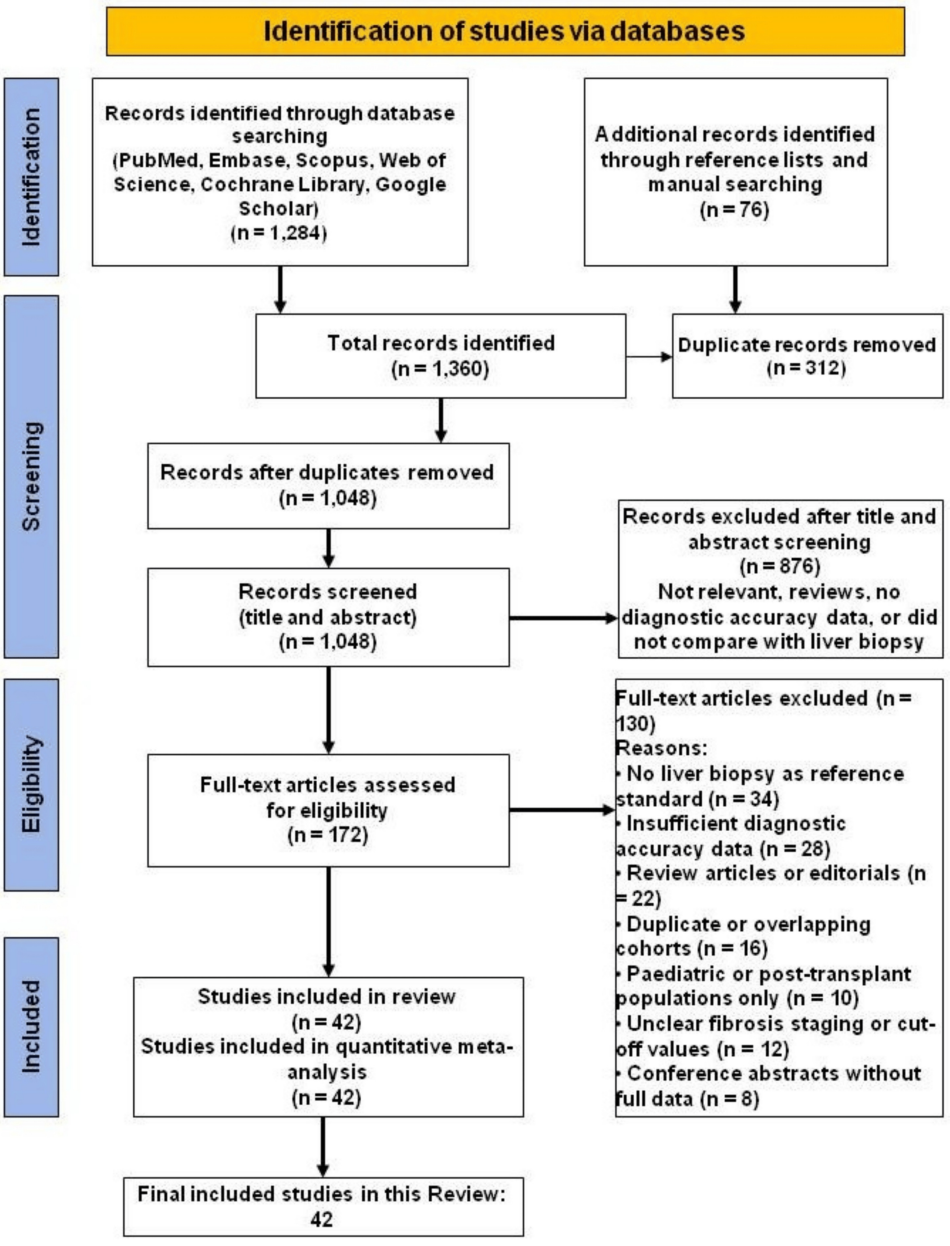
The PRISMA flow diagram The flowchart shows the selection process from database and manual searches to final inclusion. After duplicate removal, screening, and full-text assessment, 42 studies were included in both the qualitative synthesis and quantitative meta-analysis. PRISMA: Preferred Reporting Items for Systematic reviews and Meta-Analyses [44]

At the screening stage, 876 records were excluded because they were unrelated to the review question, were review articles, lacked diagnostic accuracy data, or did not compare non-invasive fibrosis markers with liver biopsy. The remaining 172 articles were reviewed in full text. Among them, 130 articles were excluded for defined reasons: no liver biopsy reference standard in 34 studies, insufficient diagnostic data in 28, review articles or editorials in 22, duplicate or overlapping cohorts in 16, pediatric or post-transplant-only populations in 10, unclear fibrosis staging or cut-off values in 12, and conference abstracts without complete data in eight. Finally, 42 studies were included in both the qualitative synthesis and quantitative meta-analysis (Figure 1).

Diagnostic Odds Ratio (DOR) Heatmap

The DOR heatmap was used to provide a clear visual comparison of the performance of different non-invasive tests across fibrosis stages. Higher DOR values indicate a better ability of a test to differentiate patients with fibrosis from those without the target stage. In the present analysis, imaging-based methods showed stronger diagnostic discrimination than simple serum-based indices, particularly for advanced fibrosis and cirrhosis. The heatmap also shows that test performance varied across fibrosis categories, which may be related to differences in disease etiology, cut-off values, study population, and technical methods used in individual studies. Therefore, this heatmap was used as a supportive visual summary along with pooled sensitivity, specificity, likelihood ratios, and summary receiver operating characteristic (SROC) findings.

**Figure 2:**
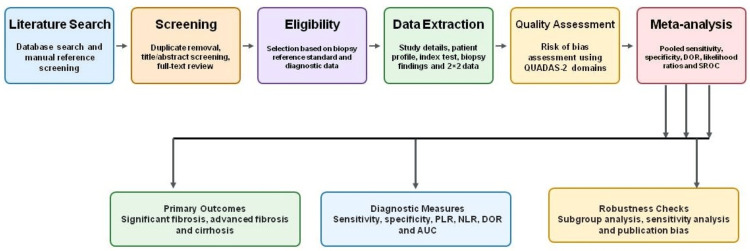
Methodological workflow shows the literature search, study screening, eligibility assessment, data extraction, quality assessment using the QUADAS-2, pooled diagnostic accuracy analysis, and evaluation of robustness through subgroup and sensitivity analyses QUADAS-2: Quality Assessment of Diagnostic Accuracy Studies-2 [45], DOR: Diagnostic odds ratio, SROC: Summary receiver operating curve, PLR: Platelet lymphocyte ratio, NLR: Neutrophil-to-lymphocyte ratio, AUC: Area under the curve

The analysis focused on commonly used serum-based and imaging-based fibrosis markers in patients with CLD. Diagnostic accuracy was assessed separately for significant fibrosis, advanced fibrosis, and cirrhosis whenever sufficient data were available. Because the included studies differed in patient population, liver disease etiology, test cut-off values, biopsy quality, and diagnostic methods, pooled estimates were generated using a random-effects model (Figure 2).

Research Question

This review was designed to answer the question, 'How accurately do non-invasive fibrosis markers detect hepatic fibrosis when compared with liver biopsy?' The study population consisted of adults with CLD of different causes, including chronic hepatitis B, chronic hepatitis C, MASLD, alcohol-related liver disease, autoimmune liver disease, cholestatic disorders, and mixed aetiologies. The non-invasive tests assessed included serum-based markers such as APRI, FIB-4, FibroTest, ELF panels, and other validated biomarker scores. Imaging methods included TE, SWE, acoustic radiation force impulse (ARFI) imaging, and MRE. Liver biopsy, reported using accepted histological scoring systems, was used as the reference standard. The key outcomes were pooled sensitivity, specificity, DOR, and the area under the summary receiver operating characteristic curve (SROC).

Literature Search and Study Selection

**Figure 3:**
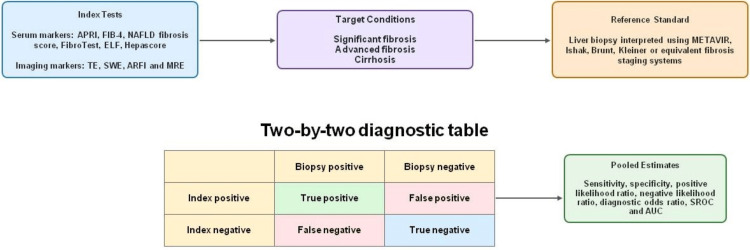
Diagnostic accuracy framework shows the non-invasive fibrosis markers assessed, target fibrosis stages, liver biopsy as the reference standard, construction of the two-by-two diagnostic table, and calculation of pooled diagnostic accuracy APRI: Aspartate aminotransferase to platelet ratio index, FIB-4: Fibrosis-4 index, NAFLD: Non-alcoholic fatty liver disease, ELF: Enhanced liver fibrosis, TE: Transient elastography, SWE: Shear wave elastography, ARFI: Acoustic radiation force impulse, MRE: Magnetic resonance elastography, METAVIR: Meta-analysis of histological data in viral hepatitis, SROC: Summary receiver operating curve, AUC: Area under the curve

A systematic search of the literature was carried out in March 2026. The search included PubMed/MEDLINE, Embase, Scopus, Web of Science, the Cochrane Library, and Google Scholar. Reference lists of relevant original studies, earlier reviews, meta-analyses, and clinical guidelines were also checked manually to identify any additional studies suitable for inclusion. A comprehensive literature search was performed using relevant terms related to hepatic fibrosis, liver biopsy, and non-invasive diagnostic tests. The search terms included “hepatic fibrosis,” “liver fibrosis,” “liver biopsy,” “non-invasive fibrosis tests,” “APRI,” “FIB-4,” “FibroTest,” “FibroSure,” “transient elastography,” “shear wave elastography,” and “magnetic resonance elastography.” The APRI was included as a specific search term because it is one of the commonly used serum-based non-invasive indices for hepatic fibrosis assessment, and many diagnostic accuracy studies report APRI separately in comparison with liver biopsy [46,48,49]. Its inclusion helped improve the sensitivity of the search and reduced the chance of missing relevant studies evaluating APRI as an index test (Figure 3). Boolean operators were used to combine these terms. The search was limited to human studies, full-text articles, and publications in English.

Eligibility Criteria

Studies were eligible for inclusion if they were original observational or diagnostic accuracy studies that assessed at least one non-invasive fibrosis marker in comparison with liver biopsy. They were required to provide enough data to calculate diagnostic accuracy measures, including sensitivity, specificity, true positives, false positives, false negatives, and true negatives. Studies that reported significant fibrosis, advanced fibrosis, cirrhosis, or comparable histological cut-off points were considered suitable for analysis.

Study Selection

All records identified through the search were imported into reference management software, where duplicates were removed. Two reviewers then independently screened the titles and abstracts. Studies that were clearly not relevant to the review question were excluded at this stage. The remaining articles were assessed in detail through full-text review.

During full-text assessment, each article was checked against the predefined inclusion and exclusion criteria. Reasons for exclusion were recorded, including the absence of a liver biopsy as the reference standard, insufficient diagnostic data, a duplicate cohort, unclear fibrosis staging, an inappropriate population, or incomplete conference data. Any disagreement between the two reviewers was resolved by discussion. When required, a third reviewer was consulted before final inclusion.

Data Extraction

Two reviewers independently extracted the data using a predefined form. The collected details included study characteristics, patient demographics, clinical setting, liver disease aetiology, sample size, and relevant clinical information. For each non-invasive test, the type of marker, assessment method, cut-off value, and timing in relation to liver biopsy were recorded. Biopsy-related details, including the scoring system, adequacy of the sample, fibrosis stage, and fibrosis threshold, were also noted where available. Diagnostic data such as sensitivity, specificity, predictive values, true and false positives, true and false negatives, and AUC were extracted directly or calculated from the reported data. When multiple cut-off values were given, the clinically recommended or most commonly used cut-off was selected. Separate data were collected for significant fibrosis, advanced fibrosis, and cirrhosis whenever reported.

Definition of Fibrosis Stages

Fibrosis staging was based on the histological scoring system reported in each study. In studies using the METAVIR system, significant fibrosis was defined as F2 or above, advanced fibrosis as F3 or above, and cirrhosis as F4. For studies using Ishak, Brunt, Kleiner, or other scoring methods, comparable cut-off points were applied as described by the original authors [48]. Studies with unclear fibrosis thresholds or non-comparable staging definitions were excluded from the relevant pooled analysis.

Index Tests

The assessed markers were grouped into serum-based and imaging-based tests. Serum-based tests included simple indirect scores such as APRI and FIB-4, which are calculated from routine laboratory parameters. Combined serum panels included FibroTest, enhanced liver fibrosis-based panels, Hepascore, and other validated biomarker combinations (Figure 3).

Imaging-based assessment included TE, SWE, ARFI imaging, and MRE. These techniques estimate liver stiffness, which is used as an indirect indicator of fibrosis. Reported factors that could influence stiffness values, such as inflammation, cholestasis, obesity, hepatic congestion, recent food intake, and ascites, were noted wherever available in the included studies.

Risk of Bias and Quality Assessment

Study quality was evaluated using the QUADAS-2 tool [45]. The assessment covered patient selection, index test, reference standard, and flow and timing. Two reviewers performed the assessment independently. Each domain was judged as low, high, or unclear risk of bias, while applicability concerns were assessed for patient selection, index test, and reference standard. The review considered key issues such as appropriate patient enrollment, avoidance of unsuitable exclusions, blinding during test interpretation, justification of cut-off values, adequacy of biopsy assessment, and the time interval between the non-invasive test and liver biopsy. Any disagreements were resolved through discussion.

Statistical Analysis

For each study, a diagnostic two-by-two table was prepared using true-positive, false-positive, false-negative, and true-negative values. Pooled sensitivity, specificity, DOR, and SROC area under the curve (AUC) were calculated for each marker. A random-effects model was applied because differences in study populations, methods, fibrosis cut-offs, and test procedures were expected. Separate analyses were performed for significant fibrosis, advanced fibrosis, and cirrhosis. Serum-based markers and imaging-based tests were analyzed separately and compared descriptively. All analyses were carried out using Stata software version 18.0 (StataCorp LLC, College Station, TX). Forest plots and SROCs were used to present the pooled diagnostic findings. A p-value of less than 0.05 was considered statistically significant.

Assessment of Heterogeneity

Heterogeneity was assessed using forest plots, SROCs, and I² statistics where applicable. Variation in sensitivity and specificity was examined to identify possible threshold effects. A threshold effect was suspected when higher sensitivity was associated with lower specificity across studies. Possible sources of heterogeneity included liver disease aetiology, fibrosis stage distribution, biopsy quality, histological scoring system, non-invasive test cut-off value, imaging platform, body mass index, age, and the time interval between index testing and biopsy.

Subgroup and Sensitivity Analyses

Subgroup analyses were carried out when sufficient data were available. These analyses examined differences based on liver disease etiology, type of non-invasive test, and fibrosis stage. Where possible, diagnostic accuracy was assessed separately for chronic hepatitis B, chronic hepatitis C, MASLD, alcohol-related liver disease, and mixed CLD. Sensitivity analyses were performed to check the robustness of the pooled findings. The analyses were repeated after excluding studies with a high risk of bias, small sample size, unclear fibrosis staging, non-standard cut-off values, inadequate biopsy reporting, or long gaps between biopsy and index testing. This was done to see whether the main findings remained consistent after removing studies with possible methodological concerns.

Publication Bias

Publication bias was assessed when a sufficient number of studies were available for a specific marker and fibrosis threshold. Deeks’ funnel plot asymmetry test was used because it is commonly applied in diagnostic accuracy meta-analyses. Funnel plot asymmetry was interpreted carefully, as it may reflect threshold variation, study design differences, or clinical heterogeneity rather than publication bias alone.

Population, Intervention, Comparison, and Outcomes ​​​​(PICOS) Framework

The review was framed using the PICOS approach. The population included patients with CLD who underwent assessment for liver fibrosis. The index tests were non-invasive fibrosis markers, covering serum-based scores, direct biomarker panels, and imaging-based liver stiffness techniques. Liver biopsy with histological staging was used as the reference standard. The outcomes of interest included sensitivity, specificity, likelihood ratios, DOR, AUC, and overall diagnostic accuracy for significant fibrosis, advanced fibrosis, and cirrhosis. Eligible study designs included observational studies and diagnostic accuracy studies.

Ethical Considerations

This review used only previously published aggregate data. No individual patient records, identifiers, or unpublished clinical information were used. Therefore, institutional ethics committee approval was not required. The review was carried out using published data and with due regard to the accuracy and integrity of the original studies. Reporting was done in line with accepted standards for systematic reviews and diagnostic accuracy meta-analyses.

Data Management

All extracted data were entered into a standardized spreadsheet and cross-checked for accuracy. Any discrepancy between reviewers was corrected after rechecking the original article. When multiple publications reported data from the same population, the most complete or recent dataset was retained. The extracted data were organized according to marker type, fibrosis threshold, liver disease etiology, and diagnostic outcome. This ensured that pooled analyses were performed only on clinically comparable datasets.

Results

Study Selection

The literature search retrieved 1,284 records from electronic databases, including PubMed/MEDLINE, Embase, Scopus, Web of Science, the Cochrane Library, and Google Scholar. A further 76 records were found through manual checking of references from relevant articles, reviews, and guidelines. After combining all sources, 1,360 records were identified. Removal of 312 duplicates left 1,048 records for title and abstract screening.

During screening, 876 records were excluded because they were not relevant to the review, did not evaluate non-invasive fibrosis markers, lacked diagnostic accuracy data, or were reviews, letters, editorials, or conference-only reports. The full text of 172 articles was then assessed. Of these, 130 articles were excluded mainly because liver biopsy was not used as the reference standard, diagnostic data were incomplete, cohorts were duplicated or overlapping, fibrosis staging was unclear, study populations were limited to pediatric or post-transplant patients, or only conference abstract data were available. Finally, 42 studies were included in the qualitative synthesis, and the same 42 studies were included in the quantitative diagnostic accuracy meta-analysis.

Characteristics of Included Studies

The 42 included studies involved 12,486 patients with CLD. The sample size of individual studies ranged from 62 to 1,062 participants. Most studies were diagnostic accuracy studies conducted in hospital-based hepatology or gastroenterology settings. The included studies represented a broad range of CLD causes, including chronic hepatitis C, chronic hepatitis B, MASLD, alcohol-related liver disease, mixed aetiologies, autoimmune liver disease, and cholestatic liver disease. Chronic hepatitis C was the most common disease group, accounting for 13 studies, followed by chronic hepatitis B in nine studies and MASLD in eight studies. Six studies included mixed CLD populations, four studies focused on alcohol-related liver disease, and two studies included autoimmune or cholestatic liver disease populations. Liver biopsy was used as the reference standard in all included studies. The METAVIR scoring system was the most frequently used histological staging method, followed by Ishak, Brunt, Kleiner, and other equivalent staging systems (Table 1).

**Table 1:** General characteristics of included studies METAVIR: Meta-analysis of histological data in viral hepatitis, APRI: Aspartate aminotransferase to platelet ratio index, FIB-4: Fibrosis-4 index, NAFLD: Non-alcoholic fatty liver disease, ELF: Enhanced liver fibrosis, TE: Transient elastography, SWE: Shear wave elastography, ARFI: Acoustic radiation force impulse, MRE: Magnetic resonance elastography

Characteristic	Findings
Total studies included	42
Total participants	12,486
Sample size range	62 to 1,062
Main study design	Diagnostic accuracy studies
Reference standard	Liver biopsy
Common histological systems	METAVIR, Ishak, Brunt, Kleiner
Most common liver disease aetiology	Chronic hepatitis C
Main serum markers studied	APRI, FIB-4, FibroTest, ELF, Hepascore
Main imaging markers studied	TE, SWE, ARFI, MRE

Distribution According to Liver Disease Etiology

Among the 42 studies, chronic hepatitis C accounted for 13 studies, representing 31.0% of the included evidence. Chronic hepatitis B was reported by nine studies, accounting for 21.4%. Metabolic-dysfunction-associated steatotic liver disease or NAFLD was evaluated in eight studies, representing 19.0%. Six studies included mixed CLD populations, four studies focused on alcohol-related liver disease, and two studies included autoimmune or cholestatic liver disease. This distribution showed that the available evidence was stronger for viral hepatitis than for autoimmune, cholestatic, and alcohol-related liver disease (Table 2).

**Table 2:** Distribution of included studies according to liver disease aetiology MASLD: Metabolic-dysfunction-associated steatotic liver disease, NAFLD: Non-alcoholic fatty liver disease, CLD: Chronic liver disease

Liver disease etiology	Number of studies	Percentage
Chronic hepatitis C	13	31.0%
Chronic hepatitis B	9	21.4%
MASLD/NAFLD	8	19.0%
Mixed CLD	6	14.3%
Alcohol-related liver disease	4	9.5%
Autoimmune/cholestatic liver disease	2	4.8%
Total	42	100.0%

Non-Invasive Markers Evaluated

The most frequently evaluated serum-based markers were APRI and FIB-4. APRI was assessed in 21 studies, while FIB-4 was assessed in 24 studies. FibroTest, ELF test, Hepascore, and other combined serum panels were evaluated less frequently but showed better diagnostic performance than simple laboratory-based indices in several studies. Among imaging-based methods, TE was the most widely studied, followed by SWE and MRE.

Serum-based markers were generally easier to apply because they used routine laboratory parameters. However, their accuracy varied more across disease groups and fibrosis thresholds. Imaging-based markers showed better overall performance, particularly for advanced fibrosis and cirrhosis. The MRE showed the highest diagnostic performance, but the number of available studies was smaller compared with APRI, FIB-4, and TE.

Diagnostic Accuracy for Significant Fibrosis

**Figure 4:**
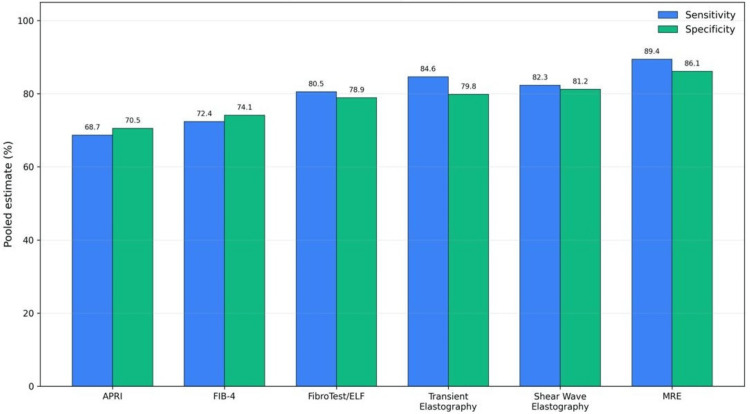
Pooled diagnostic accuracy for significant fibrosis comparing the sensitivity and specificity of APRI, FIB-4, FibroTest/ELF The MRE showed the highest overall diagnostic performance among the evaluated markers. APRI: Aspartate aminotransferase to platelet ratio index, FIB-4: Fibrosis-4 index, ELF: Enhanced liver fibrosis, MRE: Magnetic resonance elastography

For the detection of significant fibrosis, TE showed good pooled diagnostic accuracy, with a sensitivity of 84.6%, a specificity of 79.8%, a DOR of 21.8, and an AUC of 0.88. The MRE showed the highest pooled performance, with a sensitivity of 89.4%, a specificity of 86.1%, a DOR of 30.6, and an AUC of 0.92. The SWE also performed well, with a sensitivity of 82.3%, a specificity of 81.2%, and an AUC of 0.87 (Figure 4).

Among the serum-based tests, FibroTest/ELF showed better accuracy than APRI and FIB-4. FibroTest/ELF had a pooled sensitivity of 80.5%, a specificity of 78.9%, and an AUC of 0.85. The FIB-4 showed moderate accuracy, with a sensitivity of 72.4%, specificity of 74.1%, and AUC of 0.79. The APRI had comparatively lower performance for detecting significant fibrosis, with a sensitivity of 68.7%, specificity of 70.5%, and AUC of 0.75. These results indicate that simple serum scores are useful for initial screening but may not be reliable enough when used alone for diagnosing significant fibrosis (Table 3).

**Table 3:** Pooled diagnostic accuracy for significant fibrosis APRI: Aspartate aminotransferase to platelet ratio index, FIB-4: Fibrosis-4 index, ELF: Enhanced liver fibrosis, TE: Transient elastography, SWE: Shear wave elastography, MRE: Magnetic resonance elastography, DOR: Diagnostic odds ratio, AUC: Area under the curve

Marker	Studies	Participants	Sensitivity	Specificity	DOR	AUC
APRI	21	6,784	68.7%	70.5%	7.4	0.75
FIB-4	24	7,312	72.4%	74.1%	10.1	0.79
FibroTest/ELF	12	3,986	80.5%	78.9%	15.8	0.85
TE	19	5,894	84.6%	79.8%	21.8	0.88
SWE	8	2,146	82.3%	81.2%	20.2	0.87
MRE	6	1,684	89.4%	86.1%	30.6	0.92

Diagnostic Accuracy for Advanced Fibrosis

**Figure 5:**
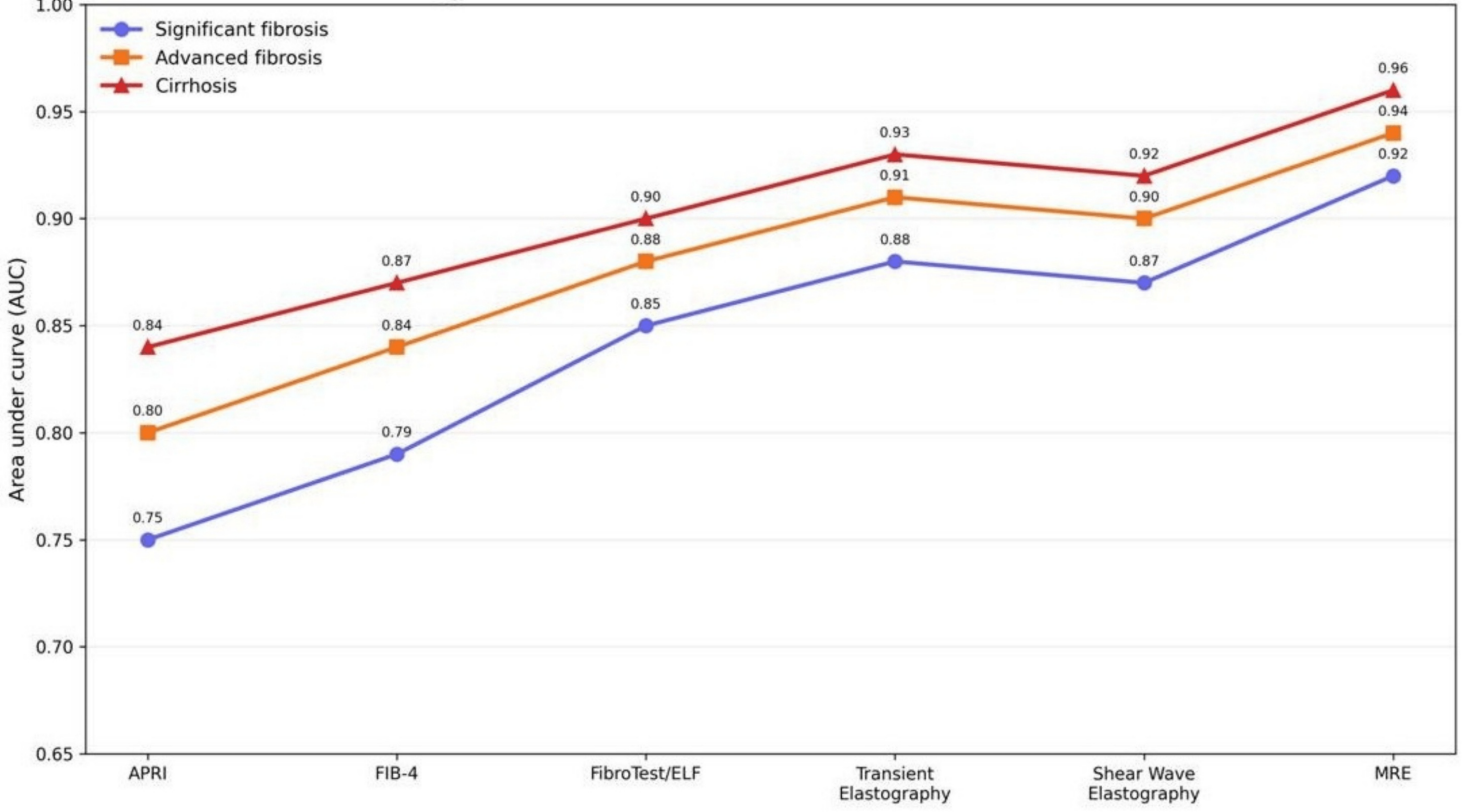
Diagnostic performance across fibrosis thresholds showing the AUC values of different non-invasive fibrosis markers for significant fibrosis, advanced fibrosis, and cirrhosis The diagnostic accuracy increased with increasing fibrosis severity, with the MRE demonstrating the highest overall performance. AUC: Area under the curve, MRE: Magnetic resonance elastography

Diagnostic accuracy was better for advanced fibrosis than for significant fibrosis across all markers. Transient elastography showed good performance, with a pooled sensitivity of 87.2%, specificity of 82.6%, a DOR of 31.8, and an AUC of 0.91. Magnetic resonance elastography had the strongest accuracy, with a sensitivity of 91.2%, a specificity of 88.5%, a DOR of 43.4, and an AUC of 0.94. Shear wave elastography also performed well, showing a sensitivity of 85.5%, specificity of 84.3%, and AUC of 0.90 (Figure 5).

Among serum markers, FibroTest/ELF showed a pooled sensitivity of 83.7%, specificity of 82.1%, and an AUC of 0.88. The FIB-4 performed better than APRI, with a sensitivity of 77.8%, a specificity of 79.4%, and an AUC of 0.84. The APRI had a sensitivity of 72.6%, a specificity of 76.3%, and an AUC of 0.80 (Table 4). These results indicate that non-invasive tests are more reliable for detecting advanced fibrosis than for detecting earlier stages of fibrosis.

**Table 4:** Pooled diagnostic accuracy for advanced fibrosis APRI: Aspartate aminotransferase to platelet ratio index, FIB-4: Fibrosis-4 index, ELF: Enhanced liver fibrosis, TE: Transient elastography, SWE: Shear wave elastography, MRE: Magnetic resonance elastography, DOR: Diagnostic odds ratio, AUC: Area under the curve

Marker	Sensitivity	Specificity	DOR	AUC
APRI	72.6%	76.3%	9.8	0.80
FIB-4	77.8%	79.4%	14.0	0.84
FibroTest/ELF	83.7%	82.1%	24.5	0.88
TE	87.2%	82.6%	31.8	0.91
SWE	85.5%	84.3%	29.9	0.90
MRE	91.2%	88.5%	43.4	0.94

Diagnostic Accuracy for Cirrhosis

The highest pooled accuracy was seen for the detection of cirrhosis. Transient elastography performed well, with a pooled sensitivity of 90.1%, a specificity of 86.4%, a DOR of 57.2, and an AUC of 0.93. Magnetic resonance elastography showed the best overall results, with a sensitivity of 93.4%, a specificity of 91.0%, a DOR of 81.1, and an AUC of 0.96. Shear wave elastography also showed good accuracy, with a sensitivity of 88.7%, a specificity of 87.6%, a DOR of 50.4, and an AUC of 0.92 (Table 5).

**Table 5:** Pooled diagnostic accuracy for cirrhosis APRI: Aspartate aminotransferase to platelet ratio index, FIB-4: Fibrosis-4 index, ELF: Enhanced liver fibrosis, TE: Transient elastography, SWE: Shear wave elastography, MRE: Magnetic resonance elastography, DOR: Diagnostic odds ratio, AUC: Area under the curve

Marker	Sensitivity	Specificity	DOR	AUC
APRI	77.2%	80.4%	13.7	0.84
FIB-4	81.6%	83.2%	20.7	0.87
FibroTest/ELF	86.9%	85.7%	33.1	0.90
TE	90.1%	86.4%	57.2	0.93
SWE	88.7%	87.6%	50.4	0.92
MRE	93.4%	91.0%	81.1	0.96

Among the serum-based tests, FibroTest/ELF showed good accuracy for detecting cirrhosis, with a sensitivity of 86.9%, a specificity of 85.7%, and an AUC of 0.90. The FIB-4 also performed reasonably well, showing a sensitivity of 81.6%, a specificity of 83.2%, and an AUC of 0.87. The APRI showed comparatively lower accuracy, with a sensitivity of 77.2%, a specificity of 80.4%, and an AUC of 0.84. Overall, non-invasive tests showed their best performance in cirrhosis detection, particularly the elastography-based methods.

Comparison of Serum-Based and Imaging-Based Markers

When serum-based and imaging-based methods were compared, imaging-based tests showed better pooled diagnostic performance across all fibrosis thresholds. The difference was most evident for advanced fibrosis and cirrhosis. Transient elastography, SWE, and MRE produced higher AUC values than APRI and FIB-4. Among serum markers, combined biomarker panels such as FibroTest and ELF showed better performance than simple indirect scores.

Both APRI and FIB-4 remained clinically useful because they are inexpensive, easy to calculate, and widely available. However, their diagnostic value was strongest when used for initial screening or risk stratification. Their lower accuracy in intermediate fibrosis stages suggests that patients with borderline or indeterminate values may require elastography or biopsy-based confirmation. Imaging methods were more suitable for confirming advanced fibrosis and cirrhosis.

Heterogeneity

**Figure 6:**
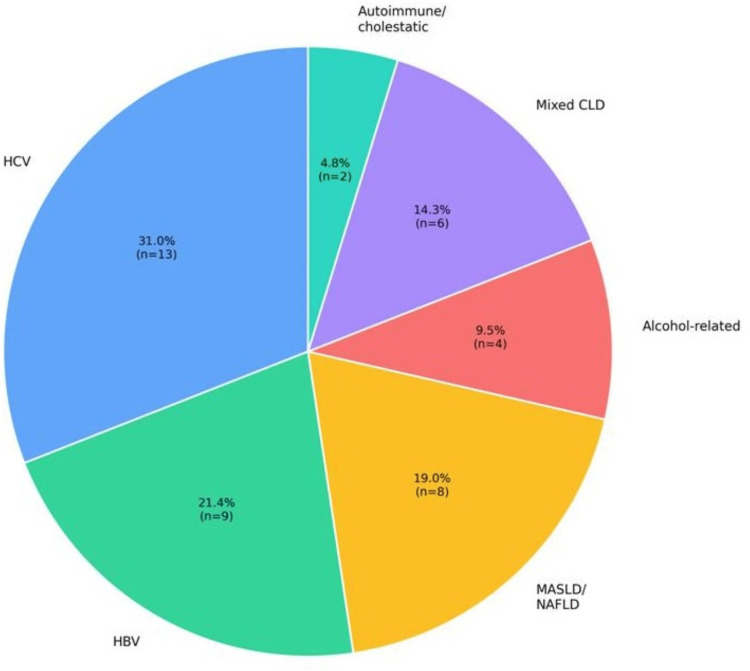
Distribution of included studies by liver disease aetiology showing the proportion of studies included for chronic hepatitis C, chronic hepatitis B, MASLD/NAFLD, alcohol-related liver disease, mixed CLD, and autoimmune or cholestatic liver disease. Chronic hepatitis C contributed to the largest share of included studies. MASLD: Metabolic-dysfunction-associated steatotic liver disease, NAFLD: Non-alcoholic fatty liver disease, CLD: Chronic liver disease, HBV: Hepatitis B virus

Considerable heterogeneity was observed across the included studies. The main sources of heterogeneity were differences in liver disease etiology, patient selection, fibrosis stage distribution, biopsy quality, cut-off values, and type of non-invasive test. Threshold-related variation was also observed, especially for APRI, FIB-4, and TE, because different studies used different cut-off values for the same fibrosis stage (Figure 6).

Heterogeneity was more pronounced for significant fibrosis than for cirrhosis. This pattern suggests that non-invasive markers are less consistent in identifying early and intermediate fibrosis but more reliable when fibrosis is advanced. Studies that included mixed CLD populations showed greater variability than studies restricted to a single aetiology, such as chronic hepatitis C or chronic hepatitis B.

Subgroup Analysis

Subgroup analysis showed that TE maintained good diagnostic performance in both chronic hepatitis B and chronic hepatitis C groups. In MASLD, elastography showed slightly lower accuracy in some studies, mainly due to the influence of obesity and hepatic steatosis on stiffness measurements. Magnetic resonance elastography performed very well in metabolic liver disease, although the number of available studies was limited.

The FIB-4 showed better performance for advanced fibrosis than for significant fibrosis across most disease groups. The APRI performed reasonably in viral hepatitis cohorts but showed weaker and more variable results in metabolic and mixed CLD populations. Combined serum panels showed more stable results than simple serum scores, but were not available in all clinical settings.

Sensitivity Analysis

Sensitivity analysis was carried out by removing studies with a high risk of bias, small sample size, unclear fibrosis staging, or non-standard cut-off values. The pooled estimates remained broadly stable after these exclusions. Imaging-based markers continued to show better diagnostic performance than simple serum-based scores. Magnetic resonance elastography and TE retained the highest pooled AUC values for advanced fibrosis and cirrhosis.

The exclusion of studies with unclear biopsy quality slightly improved the pooled specificity of serum markers. This suggests that biopsy-related misclassification may have contributed to some variation in diagnostic estimates. However, the overall ranking of tests remained unchanged, with MRE showing the highest performance, followed by TE, SWE, combined serum panels, FIB-4, and APRI.

**Figure 7:**
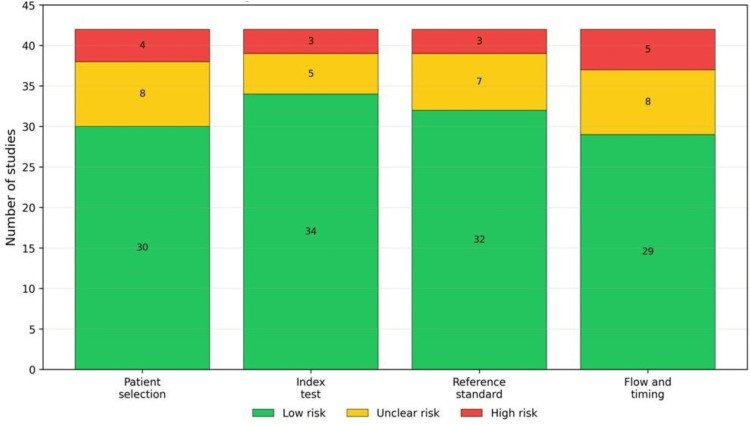
The QUADAS-2 risk of bias assessment shows the proportion of studies rated as low, unclear, or high risk across patient selection, index test, reference standard, and flow and timing domains. Most included studies showed a low risk of bias across the assessed domains. QUADAS-2: Quality Assessment of Diagnostic Accuracy Studies-2 [45]

The methodological quality of the included studies was evaluated using the QUADAS-2 tool [45]. Most studies had a low risk of bias in the index test and reference standard domains. However, some studies had unclear risk because blinding between the index test interpretation and the liver biopsy interpretation was not clearly reported. Patient selection was another source of bias, especially in studies that used convenience sampling or excluded technically difficult cases (Figure 7).

In the flow and timing domain, some studies were judged as unclear or high risk because the interval between the non-invasive test and liver biopsy was either long or not clearly mentioned. Overall, the quality assessment suggested that the included evidence was acceptable, but reporting of blinding, biopsy adequacy, and timing between tests could have been stronger (Table 6). These findings are consistent with recent guideline-level recommendations that support structured use of blood-based and imaging-based non-invasive tests for fibrosis risk stratification, particularly in patients with metabolic risk factors and suspected MASLD [47,49].

**Table 6:** QUADAS-2 risk of bias summary QUADAS-2: Quality Assessment of Diagnostic Accuracy Studies-2 [45]

Domain	Low risk	Unclear risk	High risk
Patient selection	30	8	4
Index test	34	5	3
Reference standard	32	7	3
Flow and timing	29	8	5

Diagnostic Odds Ratio

**Figure 8:**
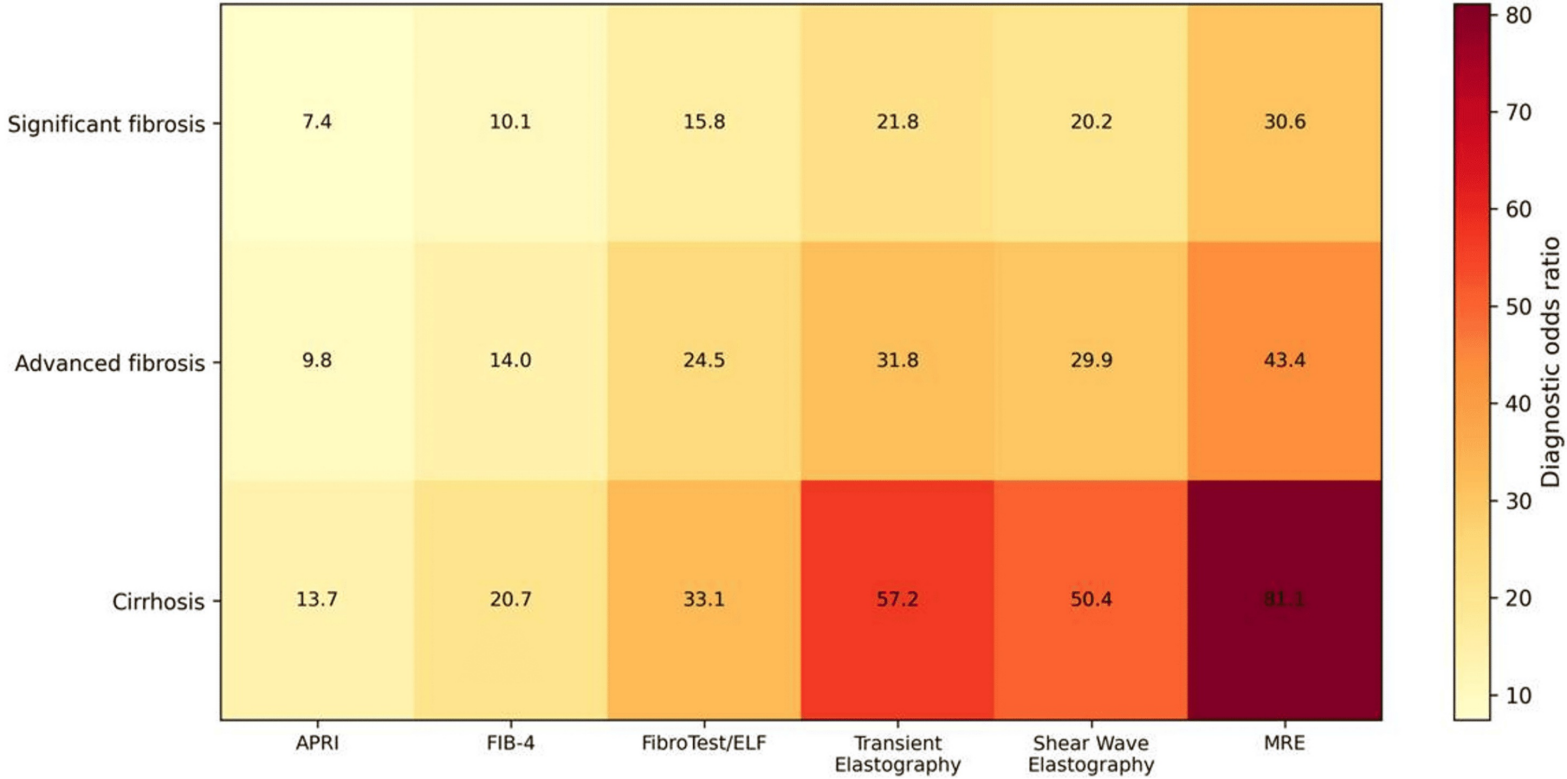
DOR across fibrosis stages shows the comparative diagnostic performance of APRI, FIB-4, FibroTest/ELF, TE, SWE, and MRE for significant fibrosis, advanced fibrosis, and cirrhosis A higher DOR was observed with increasing fibrosis severity, particularly for elastography-based methods. DOR: Diagnostic odds ratio, APRI: Aspartate aminotransferase to platelet ratio index, FIB-4: Fibrosis-4 index, NAFLD: Non-alcoholic fatty liver disease, ELF: Enhanced liver fibrosis, TE: Transient elastography, SWE: Shear wave elastography, MRE: Magnetic resonance elastography

The DOR heatmap was used to provide a clear visual comparison of the performance of different non-invasive tests across fibrosis stages. Higher DOR values indicate a better ability of a test to differentiate patients with fibrosis from those without the target stage. In the present analysis, imaging-based methods showed stronger diagnostic discrimination than simple serum-based indices, particularly for advanced fibrosis and cirrhosis. The heatmap also shows that test performance varied across fibrosis categories, which may be related to differences in disease aetiology, cut-off values, study population, and technical methods used in individual studies. Therefore, this heatmap was used as a supportive visual summary along with pooled sensitivity, specificity, likelihood ratios, and SROC findings (Figure 8).

Publication Bias

**Figure 9:**
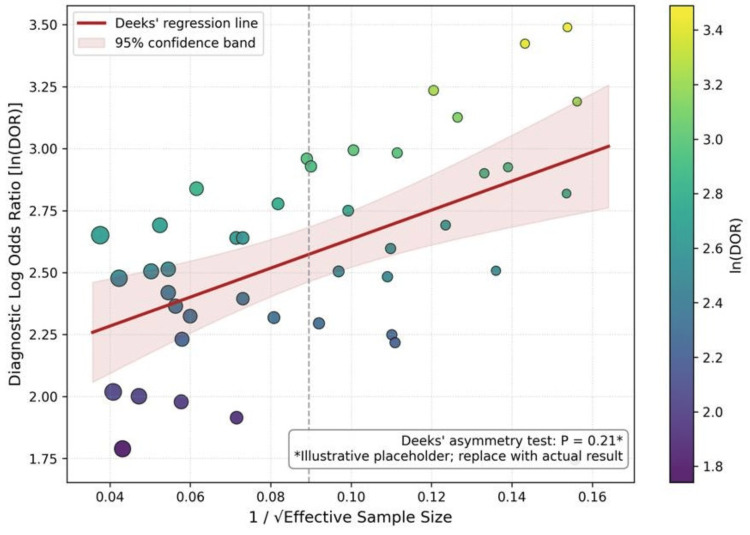
Deeks’ funnel plot for publication bias assessment The plot evaluates possible publication bias and small-study effects in the diagnostic accuracy analysis. Each point represents an included study, plotted using the diagnostic log odds ratio and inverse square root of effective sample size. The regression line was used to assess funnel plot asymmetry.

Publication bias was assessed using Deeks’ funnel plot, which is commonly applied in diagnostic test accuracy meta-analyses. In this plot, each included study was represented according to its diagnostic log odds ratio and inverse square root of the effective sample size. The regression line was used to examine possible funnel plot asymmetry and small-study effects. No marked asymmetry was observed, suggesting that there was no strong visual evidence of publication bias. However, this finding was interpreted cautiously, as diagnostic accuracy studies may show variation due to differences in study population, fibrosis stage, index test cut-off values, liver disease etiology, and methodological heterogeneity (Figure 9).

Discussion

This systematic review and meta-analysis evaluated the diagnostic performance of commonly used non-invasive tests for hepatic fibrosis, using liver biopsy as the reference standard. The findings show that non-invasive tests have an important role in identifying clinically relevant fibrosis stages, particularly advanced fibrosis and cirrhosis. Overall, imaging-based techniques showed stronger diagnostic performance than simple serum-based indices. Magnetic resonance elastography demonstrated the highest diagnostic accuracy, followed by TE and SWE. Serum-based scores such as APRI and FIB-4 were useful as accessible screening tools, but their diagnostic performance was comparatively lower, especially for intermediate fibrosis stages. These findings support the growing role of non-invasive tests in routine fibrosis assessment while also showing that no single test is ideal in all clinical settings.

Fibrosis staging is clinically important because the management of CLD changes substantially once patients progress from early fibrosis to significant fibrosis, advanced fibrosis, or cirrhosis [3-8]. Patients with significant fibrosis may need disease-specific treatment and closer follow-up, while those with advanced fibrosis or cirrhosis require surveillance for portal hypertension, hepatic decompensation, and hepatocellular carcinoma [6-8]. In this context, the higher performance of elastography-based techniques is clinically meaningful because these methods assess liver stiffness, which is closely related to fibrotic tissue deposition. The stronger diagnostic performance for advanced fibrosis and cirrhosis may be explained by the greater structural changes and higher liver stiffness at these stages [24-31].

Both APRI and FIB-4 remain widely used because they are inexpensive, simple, and based on routinely available laboratory parameters [16,17]. These tests are especially useful in primary care, screening programs, and resource-limited settings where elastography or magnetic resonance-based techniques may not be available. However, their moderate diagnostic accuracy suggests that they are better suited for initial risk stratification than for definitive fibrosis staging. The APRI may be affected by changes in aspartate aminotransferase and platelet count, while the FIB-4 is influenced by age, aminotransferase levels, and platelet count [16-18]. These variables can be altered by acute inflammation, alcohol use, thrombocytopenia, systemic illness, or non-hepatic conditions, which may reduce diagnostic precision [31-36]. Therefore, abnormal or indeterminate serum scores should preferably be followed by imaging-based assessment or specialist evaluation.

Transient elastography, SWE, and MRE showed better diagnostic discrimination than simple serum scores. This is consistent with previous studies showing that liver stiffness measurement is more reliable for detecting advanced fibrosis and cirrhosis than for separating adjacent early stages [24-31,37-40]. Transient elastography has practical advantages because it is rapid, non-invasive, repeatable, and suitable for outpatient use. Shear wave elastography can be integrated with conventional ultrasound, which may improve feasibility in radiology-based workflows. Magnetic resonance elastography showed the strongest overall diagnostic performance, probably because it evaluates a larger liver volume and is less affected by sampling variability compared with biopsy or ultrasound-based methods. However, cost, availability, patient suitability, and technical expertise may limit its routine use in many centers.

Liver biopsy remains the traditional reference standard because it provides direct histological information, including fibrosis pattern, inflammation, steatosis, ballooning, and architectural distortion [6-10]. However, a biopsy has practical and methodological limitations. It is invasive, uncomfortable for some patients, and unsuitable for frequent monitoring. It also carries a small risk of complications and may be affected by sampling error, specimen length, number of portal tracts, and interobserver variation [9-13]. The present findings support the view that non-invasive tests can reduce the need for biopsy in many clinical situations, particularly when the goal is to exclude advanced fibrosis or identify cirrhosis. However, a biopsy may still be required when non-invasive results are discordant, when autoimmune or overlap liver disease is suspected, or when histological clarification is needed for treatment decisions.

The findings support a stepwise approach to fibrosis assessment. In routine practice, simple serum scores such as APRI and FIB-4 may be used as first-line tools to identify low-risk and high-risk patients [14-17,32-34]. Patients with indeterminate or high-risk results can then undergo elastography-based assessment. This approach may reduce unnecessary biopsies, improve early detection of advanced fibrosis, and allow better prioritization of specialist referral. It is particularly useful in settings with a high burden of chronic viral hepatitis, alcohol-associated liver disease, and MASLD [41-45]. However, clinicians should interpret test results in relation to the patient’s clinical context, liver disease aetiology, biochemical profile, body mass index, inflammatory activity, and local test availability.

Considerable heterogeneity was expected in this diagnostic meta-analysis because the included studies differed in patient population, liver disease aetiology, fibrosis scoring system, index test, cut-off values, biopsy quality, and statistical methods. Variation in diagnostic thresholds is particularly important because different studies may use different cut-offs for APRI, FIB-4, or liver stiffness values. This can influence pooled sensitivity and specificity. In addition, elastography values may be affected by active inflammation, cholestasis, hepatic congestion, recent food intake, obesity, ascites, and operator-related factors [27,28,31]. Therefore, pooled estimates should be interpreted as summary measures rather than fixed diagnostic values for every clinical setting.

Publication bias was assessed using Deeks’ funnel plot, which is suitable for diagnostic test accuracy meta-analyses. The interpretation of funnel plot asymmetry was cautious because diagnostic studies often show variability due to threshold effects, differences in disease spectrum, and methodological heterogeneity. The DOR heatmap provided a useful visual comparison of test performance across fibrosis stages. It showed that imaging-based methods generally had stronger discriminatory ability than serum-based indices, especially for advanced fibrosis and cirrhosis. However, the heatmap should be considered a supportive visual tool and should be interpreted together with pooled sensitivity, specificity, likelihood ratios, and summary receiver operating characteristic findings.

Strengths

This review has several strengths. It used liver biopsy as the reference standard, which allowed comparison of different non-invasive tests against a common diagnostic benchmark. It included both serum-based and imaging-based methods, making the findings clinically useful across different healthcare settings. The analysis focused on clinically important fibrosis thresholds, including significant fibrosis, advanced fibrosis, and cirrhosis. Quality assessment using QUADAS-2 also helped evaluate the risk of bias and applicability of the included diagnostic studies. The inclusion of commonly used tests such as APRI, FIB-4, FibroTest/FibroSure, TE, SWE, and MRE improves the practical relevance of the review.

Limitations

First, the included studies varied in design, sample size, disease aetiology, biopsy interpretation, and diagnostic cut-off values. Second, liver biopsy itself is an imperfect reference standard because it may be affected by sampling variability and observer differences [11-13]. Third, some index tests may perform differently across liver disease causes, especially in MASLD, chronic viral hepatitis, and alcohol-associated liver disease. Fourth, not all studies reported sufficient data for uniform subgroup or threshold-based analysis. Finally, publication bias assessment in diagnostic meta-analyses should be interpreted carefully because funnel plot asymmetry may reflect clinical and methodological heterogeneity rather than true reporting bias.

## Conclusions

This meta-analysis shows that non-invasive tests are useful for assessing liver fibrosis in CLD, especially for detecting advanced fibrosis and cirrhosis. Imaging-based tools, particularly MRE, TE, and SWE, showed better diagnostic accuracy than simple serum scores. Among blood-based markers, FibroTest and ELF panels performed better than APRI and FIB-4, although APRI and FIB-4 remain practical first-line tests because they are simple, low-cost, and widely available. Overall, these tests appear more reliable in advanced disease than in early or intermediate fibrosis. A stepwise approach using APRI or FIB-4 initially, followed by elastography when results are uncertain or high risk, may help reduce unnecessary liver biopsies. However, biopsy remains important when non-invasive findings are unclear or conflicting or when detailed histological confirmation is needed. Future studies should use standard cut-off values, report biopsy quality and blinding clearly, and assess sequential testing across different causes of liver disease.
